# Matching supplementary motor area-primary motor cortex paired transcranial magnetic stimulation improves motor dysfunction in Parkinson’s disease: a single-center, double-blind randomized controlled clinical trial protocol

**DOI:** 10.3389/fnagi.2024.1422535

**Published:** 2024-08-01

**Authors:** Xiaoshun Tang, Zhexue Huang, Guangyue Zhu, Haoyuan Liang, Hui Sun, Yu Zhang, Yalin Tan, Minglong Cui, Haiyan Gong, Xijin Wang, Yu-Hui Chen

**Affiliations:** ^1^Department of Neurology, Tongji Hospital, Tongji University, Shanghai, China; ^2^Department of Rehabilitation, Shanghai University of Traditional Chinese Medicine, Shanghai, China; ^3^School of Life Science and Technology, ShanghaiTech University, Shanghai, China

**Keywords:** Parkinson’s disease, transcranial magnetic stimulation, SMA-M1 paired stimulation, motor dysfunction, clinical trial

## Abstract

**Background:**

Non-invasive neuroregulation techniques have been demonstrated to improve certain motor symptoms in Parkinson’s disease (PD). However, the currently employed regulatory techniques primarily concentrate on stimulating single target points, neglecting the functional regulation of networks and circuits. The supplementary motor area (SMA) has a significant value in motor control, and its functionality is often impaired in patients with PD. The matching SMA-primary motor cortex (M1) paired transcranial magnetic stimulation (TMS) treatment protocol, which benefits patients by modulating the sequential and functional connections between the SMA and M1, was elucidated in this study.

**Methods:**

This was a single-center, double-blind, randomized controlled clinical trial. We recruited 78 subjects and allocated them in a 1:1 ratio by stratified randomization into the paired stimulation (*n* = 39) and conventional stimulation groups (*n* = 39). Each patient underwent 3 weeks of matching SMA-M1 paired TMS or sham-paired stimulation. The subjects were evaluated before treatment initiation, 3 weeks into the intervention, and 3 months after the cessation of therapy. The primary outcome measure in this study was the Unified Parkinson’s Disease Rating Scale III, and the secondary outcome measures included non-motor functional assessment, quality of life (Parkinson’s Disease Questionnaire-39), and objective assessments (electromyography and functional near-infrared spectroscopy).

**Discussion:**

Clinical protocols aimed at single targets using non-invasive neuroregulation techniques often improve only one function. Emphasizing the circuit and network regulation in PD is important for enhancing the effectiveness of TMS rehabilitation. Pairing the regulation of cortical circuits may be a potential treatment method for PD. As a crucial node in motor control, the SMA has direct fiber connections with basal ganglia circuits and complex fiber connections with M1, which are responsible for motor execution. SMA regulation may indirectly regulate the function of basal ganglia circuits. Therefore, the developed cortical pairing stimulation pattern can reshape the control of information flow from the SMA to M1. The novel neuroregulation model designed for this study is based on the circuit mechanisms of PD and previous research results, with a scientific foundation and the potential to be a means of neuroregulation for PD.

**Clinical trial registration**: ClinicalTrials.gov, identifier [ChiCTR2400083325].

## Introduction

Parkinson’s disease (PD) is a neurodegenerative disease characterized by the early and significant loss of dopaminergic neurons in the substantia nigra pars compacta (SNpc). Dopamine deficiency in the basal ganglia leads to motor disorders typical of PD, including tremors, bradykinesia, stiffness, and postural instability (Kalia and Lang, 2015). The pathology of PD is also related to the formation of protein aggregates containing α-synuclein in the substantia nigra neurons, known as Lewy bodies and Lewy neurites (Gibb and Lees, 1988). In PD, the neurodegenerative process and the formation of Lewy bodies extend beyond the confines of dopaminergic neurons, manifesting in the serotonergic, noradrenergic, and cholinergic systems (Dauer and Przedborski, 2003). Brain neural circuits associated with these systems are involved in the development of PD symptoms, including the “dimmer switch model” related to tremor, which focuses on the activity in the basal ganglia circuit and the cerebello-thalamo-cortical circuit (CTC circuit), as well as the interaction between these two important circuits. According to the model, brain activities related to Parkinsonian tremors originate in the basal ganglia and are then transmitted to the CTC circuit, where they maintain and amplify the tremor rhythm. In summary, the mechanisms underlying PD are complex and interdependent (Zhong et al., 2022). With an estimated global prevalence of 6.1 million individuals in 2016, PD constitutes a significant burden on healthcare systems worldwide and is projected to escalate substantially by 2040 (Dorsey et al., 2018). Current therapeutic strategies for PD primarily aim to alleviate symptoms and delay disease progression as much as possible. The most fundamental and common treatment method for managing PD is pharmacological intervention, including levodopa, dopamine agonists, monoamine oxidase inhibitors, catechol-O-methyltransferase inhibitors, and anticholinergic drugs (Connolly and Lang, 2014). However, prolonged use of these medications often causes motor complications, including dyskinesia and motor fluctuations, necessitating alternative therapeutic approaches (Olanow et al., 2009). Surgical interventions, notably deep brain stimulation (DBS), may offer symptom relief in patients with PD refractory to pharmacotherapy (Weaver et al., 2009). DBS modulates aberrant neural activity, albeit with inherent surgical risks and device-related complications, by targeting specific brain regions, including the subthalamic nucleus and globus pallidus interna (Bronstein et al., 2011).

Transcranial magnetic stimulation (TMS) has emerged as a promising non-invasive neuromodulatory technique for neurological disorders (Hallett, 2007). TMS modulates neuronal excitability by delivering magnetic pulses to targeted cortical regions, offering therapeutic potential with fewer procedural risks than surgical intervention (Lefaucheur et al., 2020). Recently, TMS has garnered interest as a potential therapeutic option for PD, providing an alternative or adjunct treatment modality to conventional pharmacotherapy and DBS. Previous studies have confirmed that high-frequency magnetic stimulation intervention in the primary motor cortex (M1) alleviates Parkinsonian motor symptoms (Chou et al., 2015). Specifically, 25 Hz treatment has demonstrated significant efficacy in patients with early-stage PD. However, it does not significantly improve dyskinetic symptoms (Kishore et al., 2014). Although repetitive transcranial magnetic stimulation (rTMS) has partially succeeded in treating Parkinsonian motor symptoms, there remains a need to investigate new rTMS stimulation paradigms to address the complex array of Parkinsonian motor symptoms (Groppa et al., 2012).

The supplementary motor area (SMA) plays a critical role in the pathophysiology of PD (Herz et al., 2014). As a key component of the motor network, the SMA is involved in the planning, initiation, and execution of voluntary movement. The SMA dysfunction can cause impairments in motor control and coordination, contributing to the hallmark motor manifestations of PD (Joundi et al., 2013). The SMA is a cortical region in the medial frontal lobe anterior to M1 (Benecke et al., 1987). It receives inputs from various cortical and subcortical structures, including the prefrontal cortex, basal ganglia, and cerebellum (Nachev et al., 2008). These connections enable SMA to integrate sensory, cognitive, and motor information to orchestrate purposeful movement sequences (Tanji and Shima, 1994). The SMA is particularly involved in the planning and initiating of complex motor tasks and sequential organization of movements (DeLong and Wichmann, 2007). Dysfunction within the SMA can disrupt the smooth execution of motor programs, leading to bradykinesia (slowness of movement) and akinesia (difficulty in initiating movement) in patients with PD (Jahanshahi et al., 2015). SMA plays a pivotal role in motor control and is involved in compensatory mechanisms in PD. Specifically, the connections within the SMA and between the SMA and cortico-subcortical motor network compensate for the impaired prefrontal-premotor cortical connections (Herz et al., 2014).

Given its pivotal role in motor control and involvement in compensatory mechanisms in PD, SMA represents a potential therapeutic target for novel interventions (Herz et al., 2014). Non-invasive brain stimulation techniques targeting the SMA, including TMS and transcranial direct current stimulation, offer adjunctive therapeutic options for PD management (Koch et al., 2009). Previous studies have demonstrated that a 5 Hz rTMS intervention can improve the Unified Parkinson’s Disease Rating Scale (UPDRS) and activities of daily living in patients with PD (Li et al., 2022).

Task-based connectivity multi-voxel pattern analysis revealed activation clusters in the right hemisphere, central prefrontal area, superior frontal gyrus, middle frontal gyrus, thalamus, and cerebellum. Consequently, conjugating SMA stimulation targets with traditional M1 interventions could potentially enhance neural regulatory efficiency (Kishore et al., 2014).

Cortico-cortical paired associative stimulation (ccPAS) protocols are innovative, non-invasive brain stimulation techniques that modulate cortical excitability and induce synaptic plasticity in specific cortical regions. Based on classical paired associative stimulation principles, pairing peripheral nerve stimulation with TMS, ccPAS protocols focus on synchronizing TMS pulses over two distinct cortical areas to induce associative plasticity within the cortico-cortical circuits. This approach holds immense potential for investigating functional connectivity between different cortical regions and developing targeted interventions for neurological and neuropsychiatric disorders (Suppa et al., 2017). Previous ccPAS studies on SMA and M1 were often conducted at a frequency of 0.2–1 Hz and a time interval of 6 ms. The effect was to increase the MEP amplitude of the M1 that is, cortical excitability (Arai et al., 2011). However, the frequency of 0.2 Hz rTMS of M1 did not significantly improve the effect of motor symptoms in patients with PD (Okabe et al., 2003). A stimulation frequency of 5 Hz can improve motor symptoms in patients with PD in both M1 and SMA (Zanjani et al., 2015; Hanoğlu et al., 2020), and this stimulation delivery mode is cross-applied rather than continuous application (Siebner et al., 2000; Hanoğlu et al., 2020; Hewedi et al., 2020). Therefore, this study is the first choice of a stimulation parameter-matching stimulus for 5 Hz. Further studies revealed that the efficacy of rTMS stimulation produced on the M1 ascends with an increase in stimulus frequency (Hewedi et al., 2020). A 15 Hz frequency is commonly employed in the clinical highest issued by the cross of stimulus frequency (Kamble et al., 2014); therefore, we optimized the M1 TMS stimulus frequency to 15 Hz. This study selected the 1:3 (5:15 Hz) stimulation mode of the SMA before M1 for intervention. This scheme met the minimum frequency of 5 Hz required to pair SMA with M1 stimulation, following the principle of pulse-time-dependent plasticity. It also achieved the optimized 15 Hz in M1 to improve the clinical treatment effect.

As mentioned above, the abnormal function of the SMA in patients with PD may not exclusively be attributed to the SMA itself but also to a dysfunction in the control circuit formed by the SMA and M1. Based on this assumption, this study devised a novel SMA-M1 paired magnetic stimulation protocol to improve the motor symptoms of patients with PD. The proposed intervention is anticipated to yield improved outcomes by regulating SMA and M1 functions and adjusting the activation sequence of the motor control circuit from the SMA to M1.

## Materials and methods

### Study design

This study adopted a single-center, double-blind, randomized, controlled clinical trial design. In total, 78 patients meeting the diagnostic criteria for PD were recruited and randomly allocated to two groups: the SMA-M1 paired TMS treatment group (experimental group) and the sham stimulation control group (control group). Furthermore, the researchers and participants were blinded to treatment allocation to ensure double binding. The experimental group received SMA-M1 paired TMS therapy, whereas the control group underwent sham stimulation therapy. Treatments were administered five times/week for 3 weeks. Regular clinical assessments, including motor function scores, quality of life assessments, and adverse event monitoring, were conducted during treatment. Finally, improvements in motor function between the two groups were compared, and the safety and tolerability of the treatments were analyzed.

### Participants

**Figure 1:**
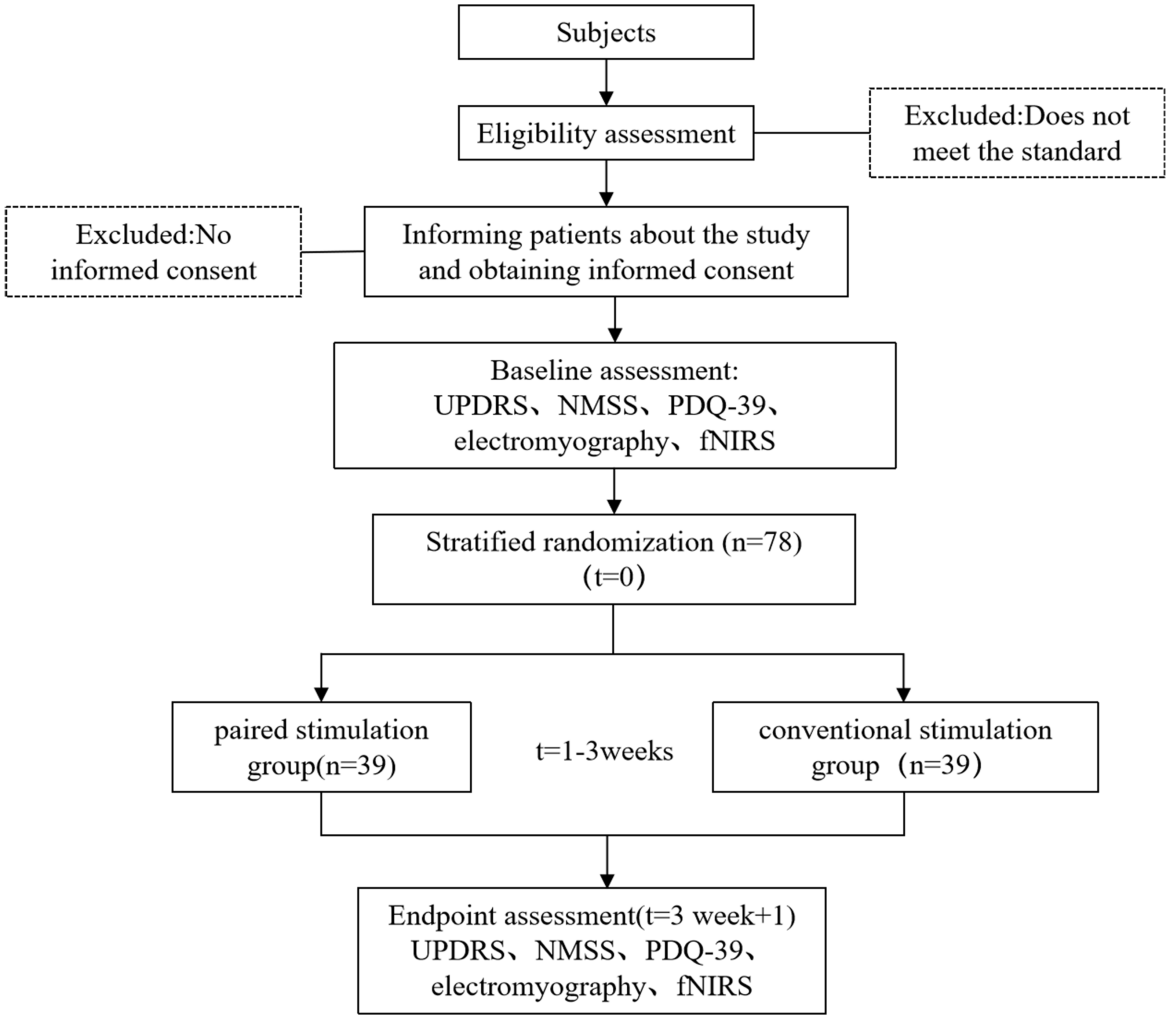
Flow chart of participants.

This study recruited participants from the Department of Neurology and Rehabilitation Medicine at Tongji Hospital, affiliated with Tongji University. Patient enlistment began on May 1, 2024. Recruitment ceased after 78 patients were enrolled. Recruitment methods primarily included advertising, physician referrals, and internet recruitment. All subjects were initially screened by a principal investigator before enrolment. Patients who met the recruitment criteria and expressed interest were individually informed of the trial details and the consent requirements. The patients signed a paper version of the informed consent form (ICF) after fully understanding the benefits and risks of this study (Figure 1).

*The inclusion criteria were as follows*: (1) PD diagnosis with motor symptoms and stable medication treatment (Hughes et al., 1992); (2) Hoehn–Yahr (H–Y) stages 1–4; (3) age 50–80 years, both genders; (4) normal cognitive function and ability to cooperate with assessment and treatment [according to the Mini-Mental State Examination (MMSE) scoring criteria] (Mitchell, 2017); (5) willingness to provide written informed consent.

*The exclusion criteria were as follows*: (1) Presence of metal implants within a 20 cm range of the stimulation site, including DBS; (2) history of epilepsy or predisposing factors for seizures [very low resting motor threshold (rMT)]; (3) currently taking medications or consuming foods that significantly impact magnetic stimulation effects, including antidepressant medications, antiepileptic drugs, central nervous system stimulants, dopaminergic medications, sedatives and sleeping pills, some analgesics, and anti-anxiety medications (Rossi et al., 2021); (4) concurrent severe illnesses that could hinder completion of treatment; (5) pregnant or lactating women; (6) patients with tumors of any kind.

### Sample size

The sample size calculation for this study was based on data from similar randomized controlled clinical trials. The main outcome measure was UPDRS III. In a similar study, the mean change in the control group was −0.05 [standard deviation (SD):1.08], and the mean change in the experimental group was −3.26 (SD:2.35) (Bhat et al., 2023). Based on our clinical experience, to ensure the trial’s reliability, we established the mean of the control group to −1, the mean of the control group to −3, and the variance of the two groups to 2.2 when calculating the sample size. The sample size was calculated using a one-tailed *t*-test and the GPower software, with the effect size set to 0.8, *α* error probability set to 0.05, and power set to 0.95. The results indicated that each group required 35 patients, with a total of 78 participants needed, considering a 10% dropout rate.

### Randomization, allocation, and blinding

This study used a randomization method known as stratified randomization. Given the significant variability in motor symptoms among patients with PD, we categorized disease severity according to the H-Y staging scale. Patients in different stages (stratified into two groups: stages 1–2 and 3–4) were randomly assigned to different groups. Randomization was performed by a third party (medical staff responsible for group assignment) not involved in the intervention or assessment. All treatment protocols were pre-set in the magnetic stimulation device (double-blind TMS trial system) by the personnel responsible for randomization. Therapists only need to follow the assigned treatment protocol numbers, eliminating the need for therapists to set prescriptions, thus ensuring blinding of the interveners. Following enrolment, the personnel responsible for randomization informed the therapists of the corresponding treatment plan numbers based on the pre-sealed codes. Intervention providers, assessors, patients, and their families were blinded to the patients’ group assignments.

All patients’ treatment protocols were pre-set in the magnetic stimulation device by the personnel responsible for randomization. Therapists strictly followed the assigned treatment protocol numbers, eliminating the need for them to establish prescriptions and ensuring blinding of the interveners. Patient assessments were recorded throughout the study. Unaware of the patients’ group assignments, the therapists performed the evaluations. In the event of anomalous data detected during data analysis or verification, the data analyst notified the principal investigator and the assessor to review the recordings for verification.

### Intervention

The study subjects were divided into two groups: paired stimulation and conventional stimulation. The paired stimulation group received the SMA-M1 paired stimulation, whereas the conventional stimulation group received high-frequency M1 stimulation. Before magnetic stimulation therapy, all subjects underwent rMT testing. Both groups undergo 1 h of rehabilitation training after stimulation, primarily focusing on limb relaxation, posture correction, and robot training. All participants underwent a three-week treatment regimen five times per week.

**Figure 2:**
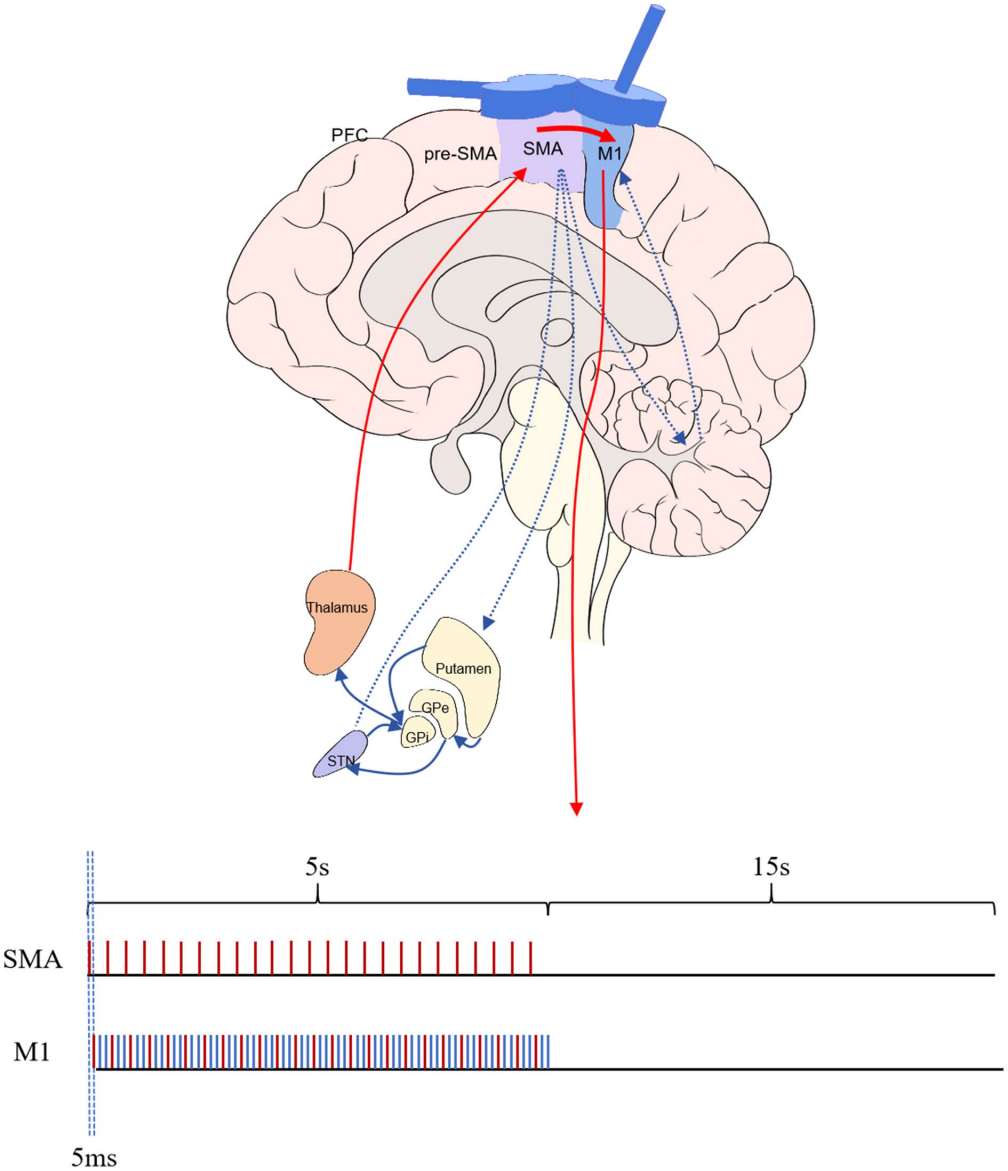
Circuit intervention mode diagram.

Our study employed an NS3000 stimulator (YIRUIDS, China) and a water-cooled child 8 type coil (YRD203F, 2.3 T), which has a center distance of two figures of 8 of 76 mm and an inner diameter of the single coil of 39 mm. It can generate a biphasic wave with a peak value of 2–3 Tesla and generate a current on both sides of the coil parallel to the longitudinal axis. This coil was capable of both dual-target and delivering sham stimulations. This study used frameless stereotaxic neuronavigation (TMS-Navigator, Localite, Sankt Augustin, Germany) to localize magnetic stimulation targets. To balance therapeutic efficiency and accuracy, we performed target localization before the initial treatment session and marked the target on a white positioning cap, using this target for stimulation in all subsequent sessions. Because some patients lack MRI data, we uniformly used a standard head phantom for registration and localization. The primary method involved using a navigation pen within the optical camera’s field of light to acquire four landmarks of the 10–20 system: the root of the nose (nasion, Nz), the external occipital protuberance (inion, Iz), and the left (LPA) and right (RPA) pre-auricular notches. The intersection of the line connecting Nz—Iz and the line connecting LPA—RPA was the spatial location of the Cz point. Then, the spatial location with the standard head phantom was registered. The navigation pen was used to determine and mark the stimulation point. Then, the positioning sphere was fixed onto the magnetic stimulation coil, the corresponding coil model in the system was selected, and the dynamic spatial position of the center point of the magnetic stimulation coil within the light field was captured. The stimulation point of the magnetic stimulation coil was aligned with the target, and once the stimulation site was confirmed, this location was marked on a white positioning cap. When stimulating the M1, the figure-of-eight coil remained tangential to the scalp, and the longitudinal axis of the handle was 45° to the nasion–inion line, which generated a current in the M1 that is 45° to the nasion–inion line. When SMA stimulation was performed, the figure-of-eight coil remained tangential to the scalp, and the longitudinal axis of the handle was 90° to the nasion–inion line, which generated a current perpendicular to the posterior–anterior current direction. When the current directions were the same, they provided stimulation consistent with the traditional figure-8 coil. When the current directions of the two were opposite, the magnetic fields canceled each other out, and no induced current was generated; that is, false stimulation was provided. The paired stimulation group was administered SMA-M1 paired stimulation, at which point the current direction in the coils of both wings of the figure-eight coil was identical. Paired stimulation was configured to trigger M1 stimulation 5 ms after SMA pulse stimulation. According to the previous studies’ results, based on the assessment of SMA and M1 separately (refer to the introduction section), the ratio of SMA and M1 paired stimulation was adjusted to 1:3, and SMA and M1 stimulation frequencies were 5 and 15 Hz, respectively. The SMA-triggered M1 stimulation formed a paired pattern, whereas the additional two M1 stimulations did not generate pairs. The stimulation intensity for both target points was 90% of the rMT, with a stimulation duration of 5 s and a rest period of 15 s, totaling 1,200 stimulation pulses. The stimulation pattern for the conventional stimulation group was similar to that of the paired stimulation group; however, at this time, the current direction in the two wings of the coil was opposite, resulting in no effective stimulation at the SMA; only the high-frequency stimulation at M1 remained unchanged (Figure 2).

### Outcome assessment

After completing the informed consent process, participants formally entered the trial protocol. Given the multitude of functional impairments in PD and the complexity of the assessment, the participants scheduled a systematic assessment with the investigator during the week preceding the intervention. Evaluations were conducted during the “on” phase of the patients. The day after concluding the four-week intervention period, the participants underwent a comprehensive assessment. For each participant, completing the second assessment marked the trial’s endpoint. Participants were informed that they could opt for a follow-up assessment free of charge within 1 week after 3 months of treatment completion. The primary components of the appraisal included basic information (gender, age, illness duration, and medication history), motor symptoms (UPDRS), non-motor functional assessment [Non-Motor Symptoms Scale (NMSS) for PD], quality of life [Parkinson’s Disease Questionnaire (PDQ-39)], and objective assessments (electromyography and functional near-infrared spectroscopy (fNIRS) brain imaging; Table 1).

**Table 1:** Recommended content for schedule of enrolment, interventions, and assessments.

	Study period						
	Enrolment & assessment	Allocation	Post-allocation			Endpoint	Follow-up
Timepoint**	−*t*_1_	0	*W*_1_	*W*_2_	*W*_3_	*W*_3_ *+ D*_1_	*M*_3_
*Enrolment*							
Eligibility screen	X						
Informed consent	X						
Allocation		X					
*Interventions*							
Paired stimulation group			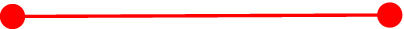				
Conventional stimulation group			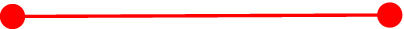				
*Assessments*							
UPDRS	X					X	X
NMSS	X					X	X
PDQ-39	X					X	X
Electromyography	X					X	X
fNIRS	X					X	X
Adverse event			X	X	X	X	X

#### Primary outcomes

The primary outcome measure of this study was UPDRS III, which comprehensively reflects the motor symptoms of patients with PD. The UPDRS is a commonly used standardized tool for evaluating the severity and symptomatology of PD. This assessment tool comprises multiple sections, including non-motor function, mood, cognition, behavior, and activities of daily living. Owing to its comprehensiveness and widespread applicability, the UPDRS has become an essential tool for assessing patients with PD in both clinical and research settings.

#### Secondary outcomes

The secondary outcome measures included the NMSS, PDQ-39, electromyography, and fNIRS brain imaging. The NMSS, primarily used to evaluate non-motor symptoms in patients with PD, aids in identifying the other benefits of SMA-M1 paired stimulation. Previous studies have indicated that even though some treatment methods do not affect motor symptoms, they may be effective for non-motor symptoms. The ultimate goal of any rehabilitation therapy is to improve the patient’s quality of life. The PDQ-39 is specifically designed to assess the quality of life of patients with PD.

**Figure 3:**
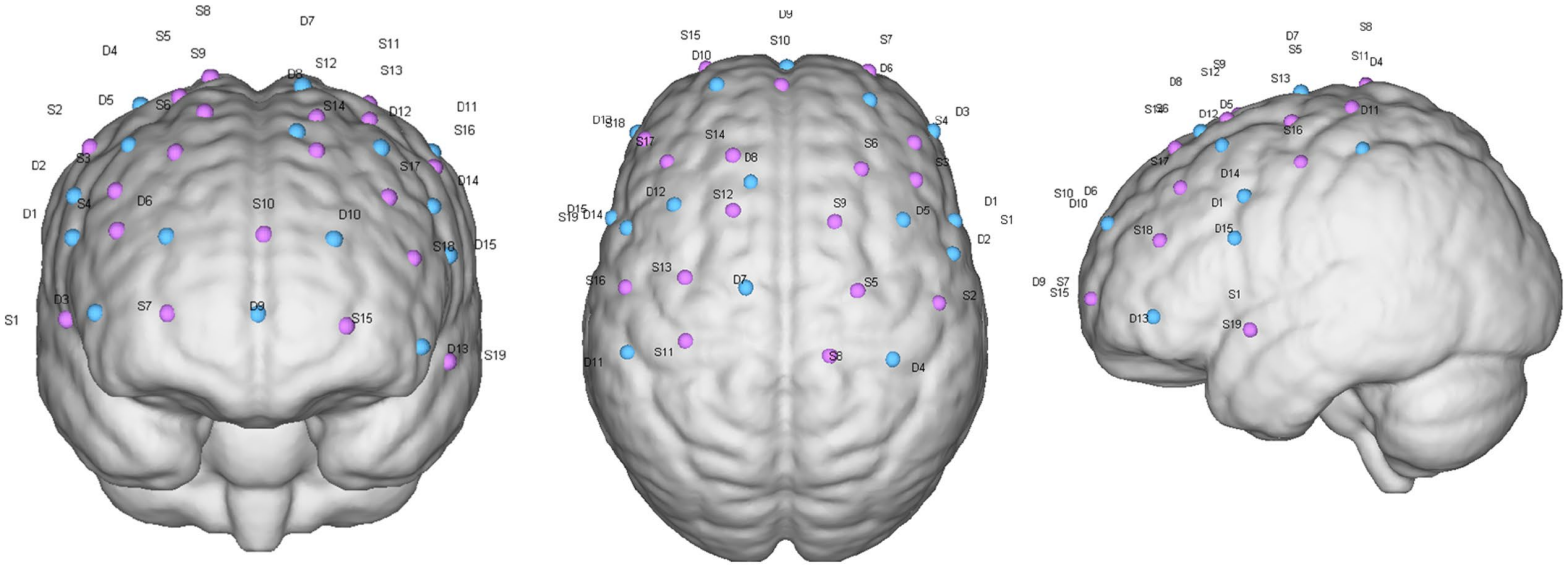
fNIRS SD arrangement.

Furthermore, combined electrophysiological and fNIRS assessments explored the conduction and circuit mechanisms of SMA-M1 paired stimulation. Motor-evoked potentials in electrophysiology can reflect the functionality of a motor circuit, which is a key circuit in motor control. The EMG assessment was matched with TMS, and MEP assessed latency, rMT, and cortical quiet period (cSP), all of which were used to assess the excitability of the corticospinal tract. Latency is the time for M1 cortical stimulation to be transmitted to the peripheral abductor pollicis brevis muscle (APB). rMT is the TMS stimulation intensity that can induce five MEP amplitudes greater than 50 μV for 10 consecutive stimulations. cSP refers to the intensity of TMS stimulation on the contralateral side when the subject actively contracts the muscle. For the cortical silent period, we established the voluntary contraction level to 20% maximum voluntary contraction (MVC). Concurrently, we used a 120% rM stimulus intensity to elicit cSP and employed subjective visual methods to determine the duration of cSP (Hupfeld et al., 2020). The duration of sTMS to the primary motor cortex caused ongoing electromyographic activity to be suppressed. Before starting the TMS assessment, an EMG electrode patch with a diameter of 25 mm was attached to the APB contralateral to the TMS stimulation cortex, with the recording electrode attached to the APB belly. The electrode was placed on the abductor pollicis brevis tendon, and the ground wire was placed inside the forearm. The patients remained quiet during the assessment period. During the TMS assessment, the coil was kept tangential to the scalp, and the current was kept at 45° to the nasion–inion line. Moreover, fNIRS reflects changes in excitability and functional connectivity in motor control-related cortices before and after treatment. The fNIRS probes were distributed over the primary motor area, premotor area, SMA, and prefrontal cortex with specific distributions (Figure 3). The near-infrared task paradigm included the resting-state and walking paradigms. The former was used to analyze the brain’s functional connectivity in patients, whereas the latter explored the relationship between cognitive function and motor cortical function during walking. The walking paradigm also included two types of tasks: single-task walking and dual-task walking. All patients were tested in the following order: resting state, single-task walking, and dual-task walking. In the resting-state paradigm, patients remained seated without engaging in any activity, with data collection lasting 8 min. Walking tasks were conducted on a straight walkway approximately 50 m long, where patients followed pre-set instructions on the computer to perform the corresponding actions. During single-task walking, the patients completed five repeated blocks, each consisting of 30 s simple walking and 30 s standing. Dual-task walking was the same as single-task walking, with the difference being that during every 30 s of walking, patients additionally underwent a pre-recorded verbal fluency test (VFT). There were 10 pre-recorded VFT questions, and one was randomly selected for playback during each 30 s of walking. Patients had to think about and answer the question while walking until the computer issued a standing instruction to stop thinking, repeated five times, with no repetition of questions in each block.

*Safety indicators* included the incidence of epilepsy and pain at the stimulation points.

### Data collection and data management

All events that caused discomfort to patients during the research process were defined as adverse events, regardless of whether these events had a causal relationship with the intervention measures. The physicians and therapists in charge of the study observed adverse events in subjects during the study, which mainly included epilepsy, pain, and dizziness based on experience. Adverse events were collected after the patient signed the ICF until the patient completed all studies or withdrew in the middle; however, adverse events occurring before the intervention were considered unrelated to the intervention plan. Adverse events that met the criteria for serious adverse events (SAEs) between the subject’s participation in the study and discharge were reported to the ethics committee as SAEs. Patients who discontinued treatment due to adverse events had their circumstances and data leading to the discontinuation recorded. SAEs in this study referred to any adverse medical events that the researchers believed were causally related to the intervention plan, resulting in any of the following conditions: likely to cause death, likely to cause serious or permanent disability, and other significant hazards identified by the clinician. SAEs occurring after the subject actively terminated informed consent or was lost to follow-up during the research process were not reported or recorded. Ensuring the accuracy and reliability of the research data is crucial for clinical studies. A series of rigorous steps were employed to maintain data quality, including data collection, verification, locking, and unblinding. The data-collection scheme and entry phase are pivotal for ensuring data integrity. Employing paper-based ICFs and specialized assessment tools for raw data acquisition minimized human error to the greatest extent possible. Two independent data entry personnel entered each ICF into the database, and discrepancies between entries were promptly rectified using software comparison tools. This process made data entry efficient and established a dependable foundation for the subsequent data analysis. The data-verification phase is critical for safeguarding data integrity and credibility. Conducted by a data administrator according to predefined verification protocols, this process incorporates various aspects, including time, logic, inclusion/exclusion criteria, and assessment results. Through meticulous verification, potential errors and inconsistencies in the data entry process were identified and corrected to ensure the data quality and reliability. The production of query resolution tables and active cooperation from researchers played an instrumental role in facilitating the data verification process.

Following the data-verification stage, a data-locking phase was implemented to safeguard data security and integrity. Once the database was audited and backed up, data locking was performed by the key researchers, statisticians, and monitors. Subsequent modifications to the database were strictly prohibited without unanimous agreement from all relevant parties, mitigating the risks of malicious tampering or inadvertent damage and ensuring the credibility and completeness of the research data.

The unblinding phase is a critical component of a clinical trial. After database locking, specialized trial statisticians conducted the statistical analysis, and the first unblinding phase revealed the treatment modalities received by the participants. After the statistical analysis reports were completed, the primary investigators compiled trial summary reports, followed by the second unblinding phase to disclose the status of the different treatment groups. The transparency and impartiality of the unblinding process are paramount for ensuring the scientific integrity and credibility of trial results.

### Statistical analysis

The incidence and severity of adverse reactions, including epilepsy, pain, and dizziness, were used for intergroup comparisons to determine whether they were related to the intervention plan. This study examined both the per-protocol and intention-to-treat set to better reflect the intervention’s efficacy.

Categorical variables, including gender and affected side, are presented as percentages, and differences between and within groups were compared using the chi-square test. Age, UPDRS, NMSS, PDQ-39, electromyography, and other continuous variables were initially tested for normality and homogeneity of variance. We employed homer2 to analyze the fNIRS data. After data collection, the Functional Connectivity NIRS (FC-NIRS) software was used to control data quality. Data that could not be processed due to abnormal fluctuations and data with a signal-to-noise ratio lower than 50 dB were removed. FC-NIRS was used for resting-state data preprocessing (mainly including data conversion, head motion correction, and band-pass filtering, as described below) and related analysis. For task status data, the MatLab-based NIRS_SPM toolbox was used for activation analysis, the Homer2 toolbox for data preprocessing and block averaging, and eigenvalues were calculated using self-written code. We employed self-written code and Statistical Package for the Social Sciences (SPSS) for statistical analysis, Origin software, and the MatLab-based BrainNet toolbox for data visualization. Data conforming to a normal distribution are presented as the mean ± SD, and differences were analyzed using paired or independent sample *t*-tests in subsequent comparisons. If the data failed to follow a normal distribution or exhibited heterogeneous variances, they are presented as medians and quartiles. Hypothesis testing was conducted using paired or nonpaired rank-sum tests. *p* = 0.05 was considered statistically significant for hypothesis testing. Considering that this study used stratified randomization and repeated measurement methods to analyze the fixed effects and random effects more accurately, this study used the linear mixed effects model to analyze the quantitative table results for overall analysis (Meteyard and Davies, 2020).

## Discussion

Functional impairment in PD is highly complex. The motor symptoms of PD include muscle stiffness, tremors, and slowing of movement, which are related to dysregulation of motor control circuits between the basal ganglia and cerebral cortex (Obeso et al., 2017). In addition to motor disorders, PD is often accompanied by non-motor symptoms, including depression, anxiety, cognitive decline, and sleep disturbances, reflecting the dysregulation of other neural circuits related to emotion, cognition, and the autonomic nervous system (Aarsland et al., 2017). Furthermore, PD progresses gradually, and the patient’s symptoms and functional impairments change over time, increasing the complexity of treatment and management (Kalia and Lang, 2015).

Clinical protocols that modulate a single target (M1 or dorsolateral prefrontal cortex) through non-invasive neuroregulation techniques often only improve one function. Emphasizing circuits and network regulation in PD is an important direction for improving the effectiveness of TMS rehabilitation. As described in a special issue of Science magazine: “No neuron is an island.” Regarding functional impairment in PD, the dysfunction of key nodes in circuits and networks is crucial for regulating PD (Foerde and Shohamy, 2011). The imbalance between the direct and indirect pathways in the basal ganglia is the core mechanism of PD (Wichmann and Delong, 2011). However, viewing PD primarily as a basal ganglia dysfunction disease is a narrow view that overlooks the impact of certain cortical circuits (the premotor cortex) on PD symptoms (Fasano et al., 2015). SMA plays a significant role in motor sequencing, temporal processing, and gait (Wu et al., 2015). Dysfunctions of the cortical and nuclear groups upstream and downstream of the basal ganglia, including the M1, premotor cortex, somatosensory cortex, and cerebellum, are direct causes of various symptoms of PD (Yugeta et al., 2010). The available non-invasive neuroregulation techniques have relatively shallow stimulation depths and cannot directly control deep nuclear groups. However, it is feasible to indirectly regulate functional cooperation between different nuclear groups by regulating cortical target areas associated with internal nuclear groups. In addition, ccPAS is a representative method of this approach (Koch et al., 2009).

Pairing the regulation of cortical circuits may be a potential method for treating PD. As a crucial node in motor control, the SMA has direct fiber connections with basal ganglia circuits and complex fiber connections with the M1 responsible for motor execution and the cerebellum, the center for balance and coordination control (Nambu, 2011). SMA regulation may indirectly regulate the function of basal ganglia circuits. Based on this, the developed cortical pairing stimulation pattern can reshape the information flow control from the SMA to M1 (Groppa et al., 2012).

In summary, the novel neuroregulation model designed for treating motor symptoms in PD was based on the circuit mechanisms of PD and previous research findings, offering a scientific foundation and the potential to be a means of neuroregulation in PD.

## Author contributions

XT: Project administration, Writing – original draft, Writing – review & editing. ZH: Data curation, Formal analysis, Visualization, Writing – review & editing. GZ: Conceptualization, Methodology, Data curation, Writing – original draft. HL: Investigation, Project administration, Writing – review & editing. HS: Project administration, Writing – review & editing. YZ: Data curation, Writing – review & editing. YT: Data curation, Writing – review & editing. MC: Investigation, Writing – review & editing. HG: Data curation, Project administration, Writing – review & editing. XW: Methodology, Visualization, Validation, Writing – review & editing. Y-HC: Funding acquisition, Methodology, Supervision, Writing – review & editing.

## References

[ref1] Aarsland D. Creese B. Politis M. Chaudhuri K. R. Ffytche D. H. Weintraub D. . (2017). Cognitive decline in Parkinson disease. Nat. Rev. Neurol. 13, 217–231. doi: 10.1038/nrneurol.2017.27, PMID: 28257128 PMC5643027

[ref2] Arai N. Müller-Dahlhaus F. Murakami T. Bliem B. Lu M.-K. Ugawa Y. . (2011). State-dependent and timing-dependent bidirectional associative plasticity in the human SMA-M1 network. J. Neurosci. 31, 15376–15383. doi: 10.1523/JNEUROSCI.2271-11.2011, PMID: 22031883 PMC6703519

[ref3] Benecke R. Rothwell J. C. Dick J. P. Day B. L. Marsden C. D. (1987). Disturbance of sequential movements in patients with Parkinson’s disease. Brain 110, 361–379. doi: 10.1093/brain/110.2.361, PMID: 3567527

[ref4] Bhat P. Kumaran S. S. Goyal V. Srivastava A. K. Behari M. (2023). Effect of rTMS at SMA on task-based connectivity in PD. Behav. Brain Res. 452:114602. doi: 10.1016/j.bbr.2023.114602, PMID: 37516209

[ref5] Bronstein J. M. Tagliati M. Alterman R. L. Lozano A. M. Volkmann J. Stefani A. . (2011). Deep brain stimulation for Parkinson disease: an expert consensus and review of key issues. Arch. Neurol. 68:165. doi: 10.1001/archneurol.2010.260, PMID: 20937936 PMC4523130

[ref6] Chou Y. Hickey P. T. Sundman M. Song A. W. Chen N. (2015). Effects of repetitive transcranial magnetic stimulation on motor symptoms in Parkinson disease: a systematic review and meta-analysis. JAMA Neurol. 72, 432–440. doi: 10.1001/jamaneurol.2014.4380, PMID: 25686212 PMC4425190

[ref7] Connolly B. S. Lang A. E. (2014). Pharmacological treatment of Parkinson disease: a review. JAMA 311, 1670–1683. doi: 10.1001/jama.2014.3654 24756517

[ref8] Dauer W. Przedborski S. (2003). Parkinson’s disease: mechanisms and models. Neuron 39, 889–909. doi: 10.1016/s0896-6273(03)00568-3 12971891

[ref9] DeLong M. R. Wichmann T. (2007). Circuits and circuit disorders of the basal ganglia. Arch. Neurol. 64, 20–24. doi: 10.1001/archneur.64.1.20 17210805

[ref10] Dorsey E. R. Elbaz A. Nichols E. Abd-Allah F. Abdelalim A. Adsuar J. C. . (2018). Global, regional, and national burden of Parkinson’s disease, 1990–2016: a systematic analysis for the Global Burden of Disease Study 2016. Lancet Neurol. 17, 939–953. doi: 10.1016/S1474-4422(18)30295-3, PMID: 30287051 PMC6191528

[ref11] Fasano A. Aquino C. C. Krauss J. K. Honey C. R. Bloem B. R. (2015). Axial disability and deep brain stimulation in patients with Parkinson disease. Nat. Rev. Neurol. 11, 98–110. doi: 10.1038/nrneurol.2014.252 25582445

[ref12] Foerde K. Shohamy D. (2011). The role of the basal ganglia in learning and memory: insight from Parkinson’s disease. Neurobiol. Learn. Mem. 96, 624–636. doi: 10.1016/j.nlm.2011.08.006, PMID: 21945835 PMC3772079

[ref13] Gibb W. R. Lees A. J. (1988). The relevance of the Lewy body to the pathogenesis of idiopathic Parkinson’s disease. J. Neurol. Neurosurg. Psychiatry 51, 745–752. doi: 10.1136/jnnp.51.6.745, PMID: 2841426 PMC1033142

[ref14] Groppa S. Oliviero A. Eisen A. Quartarone A. Cohen L. G. Mall V. . (2012). A practical guide to diagnostic transcranial magnetic stimulation: report of an IFCN committee. Clin. Neurophysiol. 123, 858–882. doi: 10.1016/j.clinph.2012.01.010, PMID: 22349304 PMC4890546

[ref15] Hallett M. (2007). Transcranial magnetic stimulation: a primer. Neuron 55, 187–199. doi: 10.1016/j.neuron.2007.06.026, PMID: 17640522

[ref16] Hanoğlu L. Saricaoglu M. Toprak G. Yılmaz N. H. Yuluğ B. (2020). Preliminary findings on the role of high-frequency (5 Hz) rTMS stimulation on M1 and pre-SMA regions in Parkinson’s disease. Neurosci. Lett. 724:134837. doi: 10.1016/j.neulet.2020.134837, PMID: 32057924

[ref17] Herz D. M. Siebner H. R. Hulme O. J. Florin E. Christensen M. S. Timmermann L. (2014). Levodopa reinstates connectivity from prefrontal to premotor cortex during externally paced movement in Parkinson’s disease. NeuroImage 90, 15–23. doi: 10.1016/j.neuroimage.2013.11.023 24269570

[ref18] Hewedi K. M. Ali A. E. Ahmed A. A. (2020). Effect of repetitive transcranial magnetic stimulation with high frequency versus low frequency on motor symptoms of Parkinson’s disease. Al-Azhar Med. J. 49, 399–410. doi: 10.21608/AMJ.2020.67801

[ref19] Hughes A. J. Daniel S. E. Kilford L. Lees A. J. (1992). Accuracy of clinical diagnosis of idiopathic Parkinson’s disease: a clinico-pathological study of 100 cases. J. Neurol. Neurosurg. Psychiatry 55, 181–184. doi: 10.1136/jnnp.55.3.181, PMID: 1564476 PMC1014720

[ref20] Hupfeld K. E. Swanson C. W. Fling B. W. Seidler R. D. (2020). TMS-induced silent periods: a review of methods and call for consistency. J. Neurosci. Methods 346:108950. doi: 10.1016/j.jneumeth.2020.108950, PMID: 32971133 PMC8276277

[ref21] Jahanshahi M. Obeso I. Rothwell J. C. Obeso J. A. (2015). A fronto-striato-subthalamic-pallidal network for goal-directed and habitual inhibition. Nat. Rev. Neurosci. 16, 719–732. doi: 10.1038/nrn4038, PMID: 26530468

[ref22] Joundi R. A. Brittain J.-S. Green A. L. Aziz T. Z. Brown P. Jenkinson N. (2013). Persistent suppression of subthalamic beta-band activity during rhythmic finger tapping in Parkinson’s disease. Clin. Neurophysiol. 124, 565–573. doi: 10.1016/j.clinph.2012.07.029, PMID: 23085388

[ref23] Kalia L. V. Lang A. E. (2015). Parkinson’s disease. Lancet 386, 896–912. doi: 10.1016/S0140-6736(14)61393-3 25904081

[ref9001] Kamble N. Netravathi M. Pal P. K. (2014). Therapeutic applications of repetitive transcranial magnetic stimulation (rTMS) in movement disorders: a review. Parkinsonism Relat Disord 20, 695–707. doi: 10.1016/j.parkreldis.2014.03.018 24726453

[ref24] Kishore A. Popa T. Balachandran A. Chandran S. Pradeep S. Backer F. . (2014). Cerebellar sensory processing alterations impact motor cortical plasticity in Parkinson’s disease: clues from dyskinetic patients. Cereb. Cortex 24, 2055–2067. doi: 10.1093/cercor/bht058 23535177

[ref25] Koch G. Brusa L. Carrillo F. Lo Gerfo E. Torriero S. Oliveri M. . (2009). Cerebellar magnetic stimulation decreases levodopa-induced dyskinesias in Parkinson disease. Neurology 73, 113–119. doi: 10.1212/WNL.0b013e3181ad5387, PMID: 19597133

[ref26] Lefaucheur J.-P. Aleman A. Baeken C. Benninger D. H. Brunelin J. Di Lazzaro V. . (2020). Evidence-based guidelines on the therapeutic use of repetitive transcranial magnetic stimulation (rTMS): an update (2014–2018). Clin. Neurophysiol. 131, 474–528. doi: 10.1016/j.clinph.2019.11.002, PMID: 31901449

[ref27] Li R. He Y. Qin W. Zhang Z. Su J. Guan Q. . (2022). Effects of repetitive transcranial magnetic stimulation on motor symptoms in Parkinson’s disease: a meta-analysis. Neurorehabil. Neural Repair 36, 395–404. doi: 10.1177/15459683221095034 35616427

[ref29] Meteyard L. Davies R. A. I. (2020). Best practice guidance for linear mixed-effects models in psychological science. J. Mem. Lang. 112:104092. doi: 10.1016/j.jml.2020.104092

[ref30] Mitchell A. J. (2017). “The Mini-Mental State Examination (MMSE): update on its diagnostic accuracy and clinical utility for cognitive disorders” in Cognitive screening instruments: a practical approach. ed. Larner A. J. (Cham: Springer International Publishing), 37–48.

[ref31] Nachev P. Kennard C. Husain M. (2008). Functional role of the supplementary and pre-supplementary motor areas. Nat. Rev. Neurosci. 9, 856–869. doi: 10.1038/nrn2478 18843271

[ref32] Nambu A. (2011). Somatotopic organization of the primate basal ganglia. Front. Neuroanat. 5:26. doi: 10.3389/fnana.2011.00026, PMID: 21541304 PMC3082737

[ref33] Obeso J. A. Stamelou M. Goetz C. G. Poewe W. Lang A. E. Weintraub D. . (2017). Past, present, and future of Parkinson’s disease: a special essay on the 200th anniversary of the shaking palsy. Mov. Disord. 32, 1264–1310. doi: 10.1002/mds.27115, PMID: 28887905 PMC5685546

[ref34] Okabe S. Ugawa Y. Kanazawa I. Effectiveness of rTMS on Parkinson’s Disease Study Group (2003). 0.2-Hz repetitive transcranial magnetic stimulation has no add-on effects as compared to a realistic sham stimulation in Parkinson’s disease. Mov. Disord. 18, 382–388. doi: 10.1002/mds.10370, PMID: 12671943

[ref35] Olanow C. W. Stern M. B. Sethi K. (2009). The scientific and clinical basis for the treatment of Parkinson disease (2009). Neurology 72, S1–S136. doi: 10.1212/WNL.0b013e3181a1d44c, PMID: 19470958

[ref36] Rossi S. Antal A. Bestmann S. Bikson M. Brewer C. Brockmöller J. . (2021). Safety and recommendations for TMS use in healthy subjects and patient populations, with updates on training, ethical and regulatory issues: expert guidelines. Clin. Neurophysiol. 132, 269–306. doi: 10.1016/j.clinph.2020.10.003, PMID: 33243615 PMC9094636

[ref37] Siebner H. R. Rossmeier C. Mentschel C. Peinemann A. Conrad B. (2000). Short-term motor improvement after sub-threshold 5-Hz repetitive transcranial magnetic stimulation of the primary motor hand area in Parkinson’s disease. J. Neurol. Sci. 178, 91–94. doi: 10.1016/s0022-510x(00)00370-1 11018700

[ref38] Suppa A. Quartarone A. Siebner H. Chen R. Di Lazzaro V. Del Giudice P. . (2017). The associative brain at work: evidence from paired associative stimulation studies in humans. Clin. Neurophysiol. 128, 2140–2164. doi: 10.1016/j.clinph.2017.08.003, PMID: 28938144

[ref39] Tanji J. Shima K. (1994). Role for supplementary motor area cells in planning several movements ahead. Nature 371, 413–416. doi: 10.1038/371413a0, PMID: 8090219

[ref40] Weaver F. M. Follett K. Stern M. Hur K. Harris C. Marks W. J. . (2009). Bilateral deep brain stimulation vs best medical therapy for patients with advanced Parkinson disease: a randomized controlled trial. JAMA 301, 63–73. doi: 10.1001/jama.2008.929, PMID: 19126811 PMC2814800

[ref41] Wichmann T. Delong M. R. (2011). Deep-brain stimulation for basal ganglia disorders. Basal Ganglia 1, 65–77. doi: 10.1016/j.baga.2011.05.001, PMID: 21804953 PMC3144572

[ref42] Wu T. Hallett M. Chan P. (2015). Motor automaticity in Parkinson’s disease. Neurobiol. Dis. 82, 226–234. doi: 10.1016/j.nbd.2015.06.014, PMID: 26102020 PMC5565272

[ref43] Yugeta A. Terao Y. Fukuda H. Hikosaka O. Yokochi F. Okiyama R. . (2010). Effects of STN stimulation on the initiation and inhibition of saccade in Parkinson disease. Neurology 74, 743–748. doi: 10.1212/WNL.0b013e3181d31e0b, PMID: 20194913

[ref44] Zanjani A. Zakzanis K. K. Daskalakis Z. J. Chen R. (2015). Repetitive transcranial magnetic stimulation of the primary motor cortex in the treatment of motor signs in Parkinson’s disease: a quantitative review of the literature. Mov. Disord. 30, 750–758. doi: 10.1002/mds.26206, PMID: 25786995

[ref45] Zhong Y. Liu H. Liu G. Zhao L. Dai C. Liang Y. . (2022). A review on pathology, mechanism, and therapy for cerebellum and tremor in Parkinson’s disease. npj Parkinsons Dis. 8:82. doi: 10.1038/s41531-022-00347-2, PMID: 35750692 PMC9232614

